# Behavior of SD-OCT Detectable Hyperreflective Foci in Diabetic Macular Edema Patients after Therapy with Anti-VEGF Agents and Dexamethasone Implants

**DOI:** 10.1155/2021/8820216

**Published:** 2021-04-13

**Authors:** Anne Rübsam, Laura Wernecke, Saskia Rau, Dominika Pohlmann, Bert Müller, Oliver Zeitz, Antonia M. Joussen

**Affiliations:** ^1^Department of Ophthalmology, Charité Universtätsmedizin Berlin, Corporate Member of Freie Universität Berlin, Humboldt-Universität zu Berlin and Berlin Institute of Health, Germany; ^2^Berlin Institute of Health (BIH), Berlin, Germany

## Abstract

**Purpose:**

Diabetic macular edema (DME) is the most common cause of blindness in the working-age population. Spectral-domain optical coherence tomography (SD-OCT) allows detection and monitoring of the edema and a detailed analysis of the retinal structure. Hyperreflective foci (HF) are small, circumscribed lesions on OCT, and their origin is yet to be determined. Our study was aimed to shed light on HF pathophysiology, by analyzing their number and location in DME patients at baseline and after therapy.

**Methods:**

A prospective, observational study on 59 eyes of 51 DME patients who were treated with antivascular endothelial growth factor (VEGF) therapy (VEGF group, *n* = 40 eyes) or dexamethasone implant (DEX group, *n* = 19). HF and hard exudates (HE) were discriminated by their appearance on fundus photographs and their size on OCT. Quantity and location of HF and HE were analyzed at baseline and after therapy.

**Results:**

DME decreased in 75% of patients in the VEGF (455.5 *μ*m vs. 380.8 *μ*m, *p* = 0.02) and in 95% of patients in the DEX group (471.6 *μ*m vs. 381.9 *μ*m, *p* = 0.007). The number of foci decreased in 62.5% of patients after anti-VEGF (130.6 vs. 111.1, *p* = 0.07) and in 68% of patients after dexamethasone injection ((123.4 vs. 94.9, *p* = 0.02) 5.1). A subgroup of 15% of eyes, all treated with anti-VEGF, showed accumulation of larger HF in outer retinal layers to visible HE during DME resolution, whereas smaller HF, found in all retinal layers, remained unchanged. There was a trend towards a dynamic shift of the foci from inner to outer retinal layers.

**Conclusion:**

The dynamic rearrangement of the small HF and their slightly greater reduction after anti-inflammatory therapy suggest inflammatory cells as their origin, whereas larger HF in the outer retinal layers correspond to microexudates. Furthermore, we found a more favourable outcome in patients with HF after treatment with dexamethasone implants compared to anti-VEGF agents.

## 1. Introduction

Diabetic macular edema (DME), a common complication of diabetes, is the primary cause of visual impairment in the working-age population of the Western world [1]. Clinical evidence indicates that there is a combination of capillary occlusion and an increased capillary permeability in DME. The ability of vascular endothelial growth factor (VEGF), to promote both vascular permeability and angiogenesis, made it a likely contributor to the vascular dysfunctions observed in DME [2, 3]. Although significant success could be demonstrated with anti-VEGF therapy, a number of limitations exist: ranging from the need for repeated intraocular injections; the socioeconomic burden of the repeated therapy; and most importantly, the fact that only approximately half of the patients show a reduction of DME after anti-VEGF therapy [4].

In the past decade, varieties of physiologic and molecular changes consistent with a role of inflammation have been found in the retinas or vitreous humor of diabetic animals and patients [5–11]. While the detailed mechanisms and their contribution to pathologies observed in diabetic retinopathy (DR) remain to be specified, these inflammatory changes seem to play a key important role in the development of DR and DME, as their inhibition has been shown to impact the development of retinal alterations in animal models of diabetes [12]. Studies using anti-inflammatory agents such as salicylates or minocycline in patients with DR gave further evidence that regulating the inflammatory response has a potentially beneficial effect, by preventing irreversible vascular and neuronal perturbations over time [13]. Dexamethasone (DEX) intravitreal implant (Ozurdex®) has been shown to be effective in the treatment of DME, with an improvement in visual acuity (VA) and a decrease in retinal thickness [14–16], even in eyes with DME refractory to anti-VEGF [15, 16]. Furthermore, as for anti-VEGF therapies [17], delay of progression and even improvement of diabetic retinopathy severity have been shown after therapy with DEX implants [18].

Recently, several authors identified baseline spectral-domain optical coherence tomography (SD-OCT) measures which can serve as biomarkers for predicting VA outcome after treatment for DME, either with DEX implants [19, 20] or anti-VEGF therapies [21–23]. The presence of subretinal fluid (SRF), continuity of the inner segment/outer segment (IS/OS) line, and the absence of HF predict better visual outcomes after treatment in eyes with DME [19, 20, 24]. Furthermore, the absence of HFs led to significantly greater VA improvement and a greater reduction in central retinal thickness (CRT) after anti-VEGF therapy [22]. HF are discrete, well-circumscribed, intraretinal lesions with greater reflectivity than the retinal pigment epithelium (RPE) band on SD-OCT [25]. The role of HF has also been investigated in several other neurovascular disorders like retinitis pigmentosa [26], retinal vein occlusion [27], morbus coats [28], and age-related macular degeneration (AMD) [29]. The exact origin of these foci is yet to be determined. They may represent subclinical features of lipoprotein extravasation after the breakdown of the inner blood-retinal barrier (BRB), which form visible hard exudates at later stages of the disease [30] and/or represent activated inflammatory cells such as microglia [31–33] or migrating RPE cells (in AMD only) [25].

The purpose of this study was to gain more insight into the pathophysiology of HF. This was done through analyzing the quantity and location of retinal HF at baseline and after therapy with one intravitreal injection of an anti-VEGF drug or a dexamethasone implant in DME patients. We hypothesized that primarily anti-inflammatory therapy by dexamethasone should influence HF differently than primarily antiangiogenic therapy by VEGF inhibition.

## 2. Material and Methods

In this noninterventional, observational, and prospective study, we recruited 59 eyes of 51 consecutive patients affected by type 2 diabetes. They were all treated at the Retina Service of the Department of Ophthalmology, Charité Universitätsmedizin Berlin, between September 2018 and June 2019. All the research and measurements adhered to the tenets of the Declaration of Helsinki; the local ethics committee approved the study. Informed consent was given by the patients via prior study enrollment.

The inclusion criteria was those aged 18 years or older with the presence of a fovea involving clinically significant DME, which necessitates DME therapy with one of the three currently available anti-VEGF agents (VEGF group): bevacizumab (Avastin®), ranibizumab (Lucentis®), aflibercept (Eylea®), or the intravitreal injection of a Dexamethasone Implant (Ozurdex®, DEX group). Patients were not randomized, but prospectively included based on their treatment (either VEGF or DEX group). Treatment modality was chosen at the discretion of the treating retina specialist.

The exclusion criteria were the presence of significant media opacities and severe visual impairment leading to low-quality SD-OCT images, planned retinal laser treatment or intraocular surgery within the follow-up period or within the past 3 months, a refractive error greater than minus 5 diopters, and a sign of any other active retinal disease in the study eye (including the presence of an epiretinal membrane or vitreomacular traction syndrome).

### 2.1. Patient Data

Baseline diagnostic procedures included a best-corrected visual acuity (BCVA) assessment in Logarithm of the Minimum Angle of Resolution (logMAR), anterior and posterior segment examination, SD-OCT (Heidelberg Spectralis, Heidelberg Engineering, Heidelberg, Germany), fluorescein angiography (FA, Heidelberg Spectralis, Heidelberg Engineering, Heidelberg, Germany), color fundus photography (CFP, Zeiss Mediatec, Jena, Germany), and OCT-angiography (OCTA, Heidelberg Engineering, Heidelberg, Germany). We performed follow-up examinations including SD-OCT and CFP 15 days after the anti-VEGF injection. Another follow-up visit with SD-OCT and CFP assessment and visual acuity testing was conducted 30 days after the intravitreal anti-VEGF injection and 30 days after the injection of the intravitreal dexamethasone implant (Ozurdex®). We chose a follow-up interval of 30 days for Ozurdex®, because the maximum reduction of the CRT after the injection is detectable 30 days after treatment [34].

### 2.2. OCT Data

Standard settings for OCT recordings were 20° × 20° volume scan, 49 sections at a distance of 122 *μ*m. Central retinal thickness (CRT) in *μ*m was automatically calculated by the device software (software version 5.1.2.0), as the distance from the RPE to the inner limiting membrane (ILM) at the highest point within a circle of 1 mm radius, centered on the fovea. We also assessed the disruption of the junction between the photoreceptor inner and outer segments (IS/OS line). An intact IS/OS line was defined as a continuous hyperreflective line on each OCT scan and a disrupted IS/OS line as the loss or discontinuity of the hyperreflective line, if present, in any of the 49 scans.

Two masked, independent investigators (LW and SR) manually counted the HF within each scan. HF were defined as discrete and well-circumscribed dots of identical reflectivity as the RPE band. The largest diameter of small HF (also referred to as hyperreflective dots) was limited to 30 *μ*m. Additional larger foci with a diameter of >30 *μ*m could be identified in a subset of patients. By utilizing these size criteria, we excluded small noise signals and large hyperreflective clumps, which are detectable as hard exudates on fundus photography. The total number of HF in each scan was counted, as well as the number of foci within the inner (from the ILM to inner nuclear layer, INL) and outer (outer plexiform layer, OPL to the RPE) retinal layers.

The eyes of the DME patients were further classified into two groups according to their pattern of edema. Focal edema was defined as a localized retinal fluid collection, mostly in the outer retinal layers (also referred to as outer retinal edema), contrary to a diffuse cystoid or noncystoid retinal thickening, derived from breakdown of the inner blood-retinal barrier. FA was performed for evaluation of retinal ischemia and vascular leakage.

### 2.3. Color Fundus Photography

Each OCT image with a hyperreflective focus was overlaid with a corresponding CFP and with the corresponding red-free image that is available for tracking on the Heidelberg Spectralis device, using the device software. For each HF on SD-OCT, we thereby ruled out other causes of hyperreflectivity such as microaneurysms, haemorrhages, cotton wool spots, retinal vessels, and hard exudates. All of which are visible on CFPs and/or near-infrared images. If any of these were found to account for the HF, these foci were excluded from the analysis.

### 2.4. Optical Coherence Tomography Angiography

**Figure 1:**
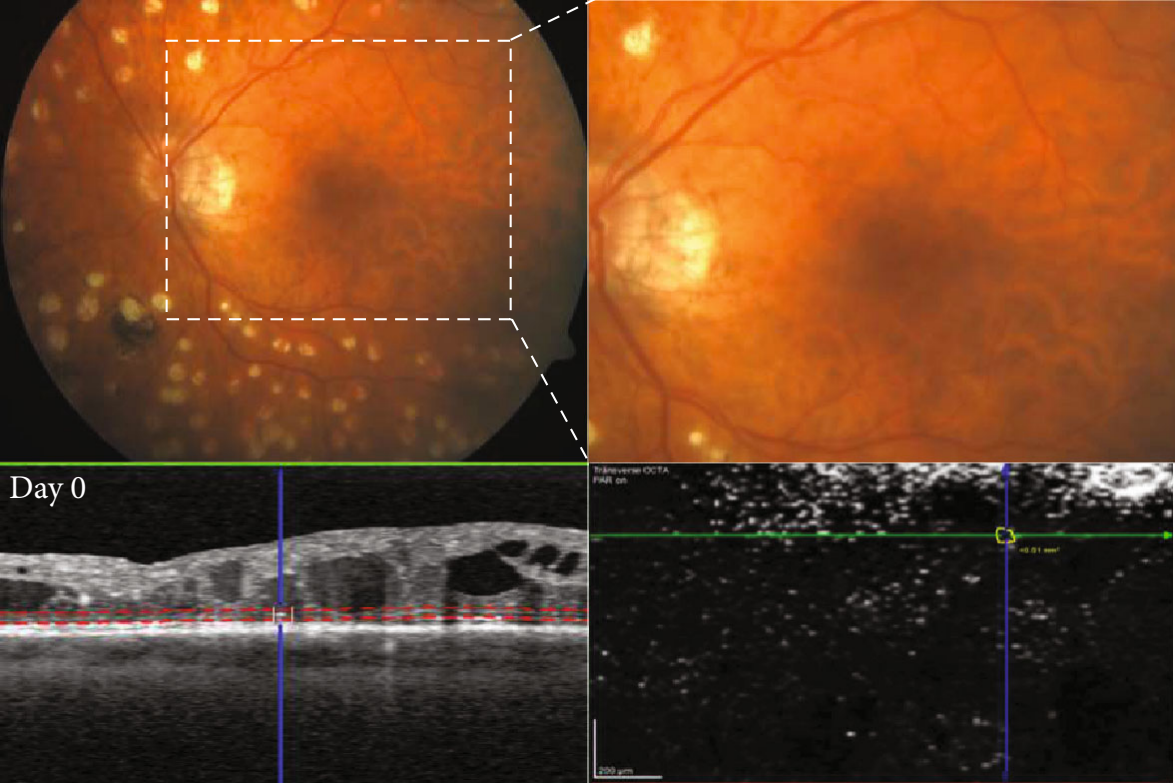
Multimodal imaging of a nonvascularized outer retinal layer (ORL) hyperreflective foci (HF). Figure shows the distribution of a HF in a 42-year-old patient with proliferative diabetic retinopathy (PDR) at baseline. Upper panel: HF in the absence of any visible HE on corresponding color fundus photographs (CFP) could be detected. Higher magnification image of the macular area marked with a dashed square. Lower panel: visualization of an outer nuclear layer HF adjacent to a cystoid space on optical coherence tomography (OCT) B-scan with manual segmentation of the vascular slabs of this region visualized as dashed red lines (left). Corresponding en face OCT-angiography (OCTA, right) image of the manually outlined vascular slab confirms, which the HF is not attached to blood vessels. HF border is outlined in yellow and area in mm^2^ is calculated by the device software.

For the OCTA examinations, 70, 000 A-Scans were made and a 15 × 15° scan angle protocol was used to gain a total of 261 B-scans, resulting in images with an axial resolution of approximately 4 *μ*m. This was within the B-scan resolution of approximately 11 *μ*m and between the B-scan resolution of approximately also 11 *μ*m. The standard OCTA viewing module 6.9.5.0 and a manual segmentation of the retinal layers was used for the evaluation of the qualitative relationship between HF on B-scan OCT images and the retinal vasculature on en face OCTA images. Instead of using the program's predefined automated segmentation, we manually selected the thickness of the vascular slab to be displayed exactly at the border of the foci to display only blood vessels adjacent to the HF. To do so, we manually moved the two depicted dashed red lines in the Angio mode B-scan, which indicate the vascular slab to be displayed at the corresponding two-dimensional en face OCTA image (with transversal slab orientation), to the border of the HF (Figure 1). The corresponding area in the en face OCTA image was then compared to the OCT scan and analyzed for the overlay of the HF with vasculature.

### 2.5. Statistical Analysis

The means ± SEM and statistically significant differences are reported. The correlation between the baseline characteristics and between the baseline and the follow-up visit were calculated with Pearsons' correlation coefficient *r* based on the assumption of a Gaussian distribution. The differences in the parameters between the baseline and final visit were evaluated using a two-tailed *t*-test. A *p* value inferior to 0,05 was considered significant. The statistical analysis, except for the kappa coefficient, was performed using GraphPad Prism (GraphPad Software, San Diego, CA, USA). Calculation of the intraclass correlation coefficient (Cohen's kappa), for evaluation of interobserver concordance of HF counting was performed using SPSS (IBM Software, Armonk, New York, USA), based on a mean rating (*k* = 2), absolute agreement, and 2-way mixed effects model.

## 3. Results

### 3.1. Baseline

The baseline characteristics of the subjects are listed in Table 1. The patients' mean age was 58.9 ± 8 years. Although DME occurred during all DR stages, more than half of the study patients had a proliferative stage. Of note, almost all DME patients with HF presented with a diffuse retinal thickening. Disruption of the IS/OS line was present in 7/59 eyes (12%) at the baseline visit. The numbers of HF ranged from 17 to 375 foci per eye at baseline (mean 131.1 ± 103). There was an excellent interrater agreement (0.938) regarding the number of counted HF.

The baseline HF number was positively correlated with the baseline CRT (*r* = 0.535, *p* < 0.001), but no significant correlation was found between the baseline presence of foci and the disruption of the IS/OS line, the DME type or DR stage at baseline, and in regard to the final number of HF and final CRT. The number of baseline HF further significantly correlated with baseline VA (*r* = 0.502, *p* < 0.001) and final VA (*r* = 0.398, *p* = 0.003). There was no difference regarding the distribution of foci and their impact on visual acuity as both, inner retinal layer and outer retinal layer HF, correlated significantly with baseline and final VA (IRL foci: *r* = 0.391, *p* = 0.003 for baseline VA and *r* = 0.351, *p* = 0.008 for final VA; ORL foci: *r* = 0.493, *p* = 0.001 for baseline VA and *r* = 0.356, *p* = 0.007 for final VA).

**Figure 2:**
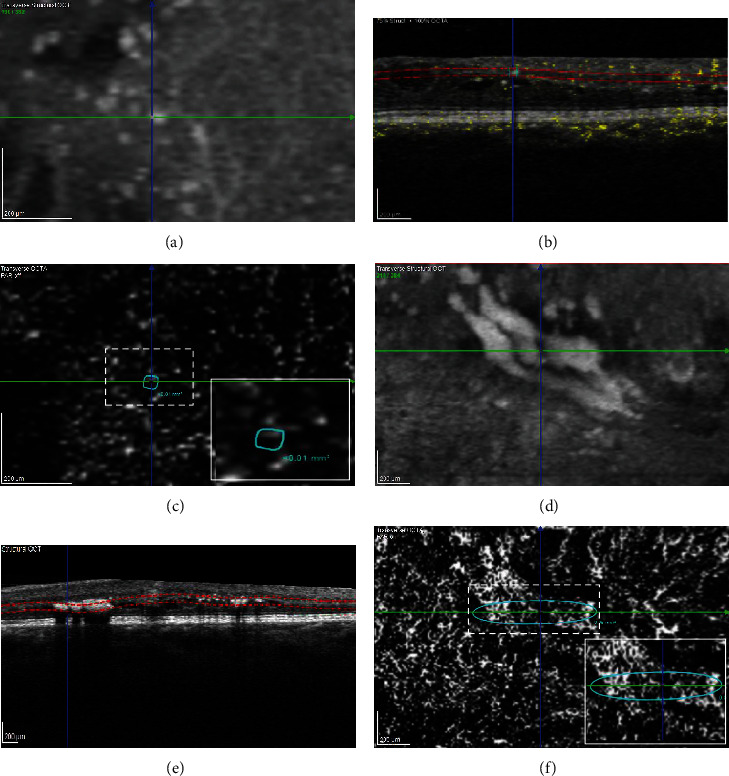
Multimodal imaging of vascularized inner retinal layer (IRL) (IRL) hyperreflective foci (HF) and hard exudates (HE). (a, d) The structural optical coherence tomography (OCT) delineates dot-like HF (a–c) and spot-like HE (d–f) in a 39-year-old patient with mild nonproliferative diabetic retinopathy (NPDR) at baseline. (b, e) B-scan with manual segmentation of the vascular slabs of the region visualized as dashed red line, depicting HF in the inner nuclear layer (INL) and HE in the outer plexiform layer (OPL). (c, f) Corresponding en- face OCT-angiography (OCTA) images of the manually outlined vascular slabs demonstrate HF and HE deposited adjacent to blood vessels. Higher magnification images of the HF and HE are marked by a dash square. Both HF and HE borders are outlined in turquoise, and area in mm^2^ is calculated by the device software.

OCT imaging demonstrated that 64% hyperreflective foci (large and small) were deposited in the outer retinal layers (OPL to RPE, Figure 1), mostly adjacent to cystoid spaces. The other 36% foci, all fine hyperreflective dots, were found to be randomly deposited in the inner retinal layers (ILM to INL, Figure 2(b)). Hard exudates were found only in the outer retinal layers (Figure 2(e)). A comparative analysis of HF on SD-OCT and corresponding OCTA scans revealed that HF were associated with blood vessels depending on their location within the retina. Whereas inner retinal layer foci were elusively attached to vessels of the superficial capillary plexus, we found outer retinal layer foci that are attached to the wall of cystoid spaces, in areas without any sign of vascularization on OCTA (Figure 2(c)). Hard exudates that are located between the IPL and the OPL were attached to vessels of the deep capillary plexus on OCTA (Figure 2(f)).

DME: diabetic macular edema; IS/OS: inner segment/outer segment; NPDR: nonproliferative diabetic retinopathy; PDR: proliferative diabetic retinopathy.

### 3.2. Follow-Up

After therapy, the overall mean CRT decreased significantly from 414.2 ± 79 *μ*m to 378.4 ± 76 *μ*m (*p* < 0.001). The overall mean number of foci decreased from 131.7 ± 103 to 115.6 ± 100 (*p* = 0.004). The mean number of HE remained unchanged after treatment (13.9 ± 18 to 13.1 ± 16, *p* = 0.223). The mean BCVA also remained unchanged (0.41 ± 0.31 to 0.41 ± 0.31, *p* = 0.929).

### 3.3. Central Retinal Thickness

**Figure 3:**
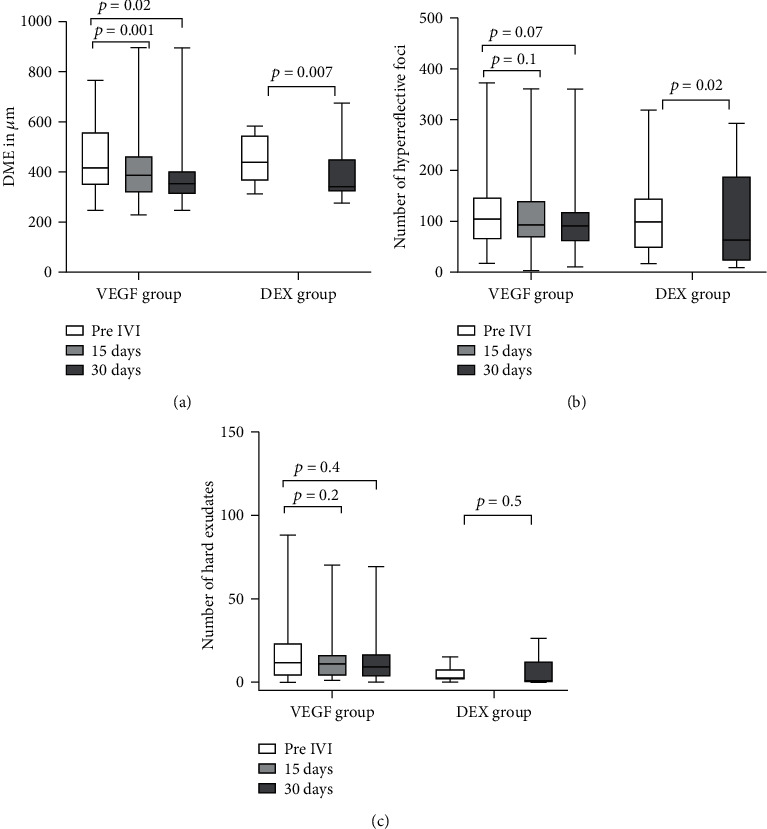
Boxplot graph representing changes in central retinal thickness (CRT), number of hyperreflective foci (HF), and number of hard exudates (HE) after anti-VEGF therapy (VEGF group) or dexamethasone implantation (DEX group) in patients with diabetic macular edema (DME). Figure shows the difference in change in (a) CRT in *μ*m (b) number of HF, and (c) number of HE for the VEGF and DEX group at baseline versus 15 or 30 days after the injection.

In the VEGF group, mean CRT decreased significantly from baseline to day 15 (*p* = 0.001) and decreased further significantly to day 30 (*p* = 0.021). In the DEX group, mean CRT also decreased significantly from baseline to day 30 (*p* = 0.007). The details are listed in Table 2 and illustrated in Figure 3. An additional excel file with all patient data, relevant for the analysis, is available as a supplemental file (see Online Resource 1).

There was no difference between patients treated with anti-VEGF and dexamethasone, regarding the reduction of retinal thickness (VEGF group: 12% reduction after 15 days, 17% after 1 month, DEX group: 22% after 1 month). Of note, the percentage of eyes, without any persistent macular fluid or a decreased edema at day 30, was higher when Ozurdex® had been injected (Table 3).

### 3.4. Hyperreflective Foci

In the VEGF group, the mean number of foci decreased only slightly after 15 days (*p* = 0.105). Then, 30 days after the injection, the mean number of foci decreased further, but without statistical significance (130.6 vs. 111.1, *p* = 0.062). In the DEX group, the mean number of foci decreased significantly after 30 days (123.4 vs. 94.9, *p* = 0.020). The details are listed in Table 2 and illustrated in Figure 3.

**Figure 4:**
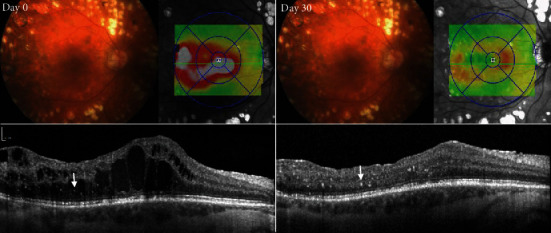
Treatment response of hyperreflective foci (HF) after dexamethasone implantation in a patient with diabetic macular edema (DME). Color fundus photograph (CFP), spectral-domain optical coherence tomography (SD-OCT) B-scan, and infrared image with Early Treatment Diabetic Retinopathy Study (EDTRS) grid depicting extent of DME (red and grey color) at baseline (left panel, day 0) and one month after dexamethasone injection (right panel, day 30) in the same patient as in Figure 1. HF are found adjacent to cystoid spaces in the outer retinal layers. After therapy, the DME resolved and the number of HF clearly decreased.

The percentage of eyes with a reduction of the number of foci at the end at one month was, as for the reduction of the DME, slightly higher in the DEX group (62.5% anti-VEGF vs. 68% for Ozurdex®, Table 2). In the VEGF group, 75% of the patients with a decreased number of foci had a decreased edema, 10% of patients presented unchanged, and 15% increased macular fluid. Of the 13/19, 68% of eyes in the DEX group had a reduced number of foci; all eyes showed a reduction in CRT, with seven patients having a dry macula 1 month after the treatment. A representative patient of the DEX group is illustrated in Figure 4.

**Figure 5:**
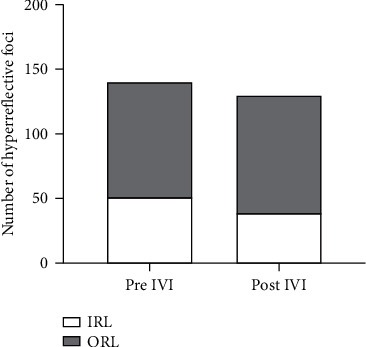
Bar graph representing changes in the distribution of hyperreflective foci (HF) in patients with diabetic macular edema (DME) treated with anti-VEGF or dexamethasone implant. Figure shows the difference in the distribution of in HF between the inner retinal layers (IRL, from the ILM to inner nuclear layer, INL) and the outer retinal layers (ORL, from the outer plexiform layer, OPL to the retinal pigment epithelium, RPE) at baseline compared to 30 days after the injection.

The fine HF were scattered throughout all retinal layers at the follow-up examination, with a trend towards a downward shift of the foci from the inner to the outer retinal layers. Before therapy, the percentage of HF in the inner retinal layers was 34%, and 66% were distributed in the outer retinal layers. After treatment, 6% of the inner retinal layer foci shifted into outer retinal layers (Figure 5).

### 3.5. Hard Exudates

**Figure 6:**
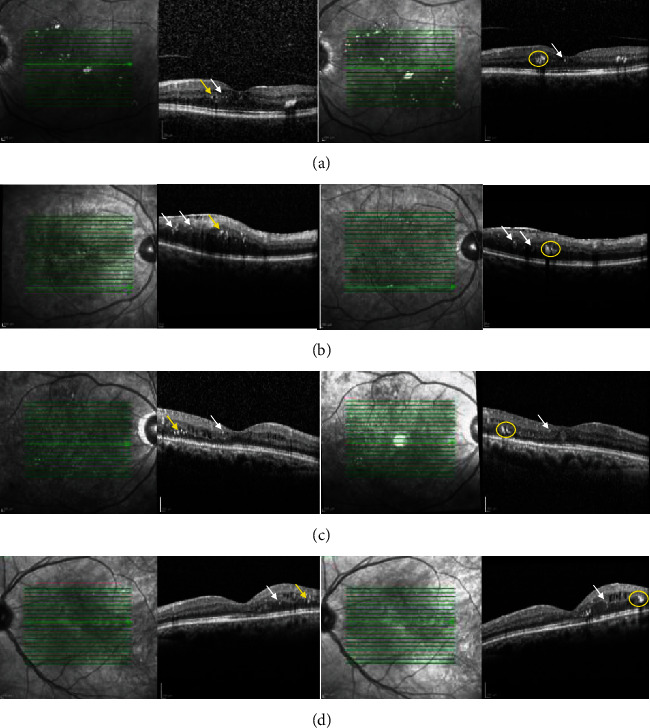
Accumulation of large hyperreflective foci (HF) to form hard exudates (HE) in patients with diabetic macular edema (DME) after anti-VEGF therapy. Figure shows infrared images and corresponding spectral-domain optical coherence tomography (SD-OCT) scans at (left panel, a–d) baseline and (right panel, a, b) two months or (right panel, c, d) one month after anti-VEGF injection. In the region of interest on SD-OCT, large HF (yellow arrows) in the outer retinal layers (outer plexiform layer (OPL) and outer nuclear layer (ONL)) increase in density and area to form HEs (yellow circles), which are visible on corresponding infrared images (right panel, a, b). Small HF (white arrows), which might resemble phagocytising macrophages, are scattered through all retinal layers and do not change in number or size after treatment.

In the VEGF group, the mean number of HE remained unchanged after treatment (17.9 ± 20 to 16.1 ± 19, *p* = 0.223 after 15 and 13.3 ± 16, *p* = 0.411 after 30 days). In the DEX group, the mean number of HE also remained unchanged after treatment (4.75 ± 5 to 5.1 ± 8, *p* = 0.562, Figure 3). A subgroup of 7/46 (15%) of eyes showed an accumulation of visible HE in the OPL during the study period. This was in an area where we detected only larger HF (>30 *μ*m) at baseline. All of these patients were treated with anti-VEGF and DME decreased in 71.4% of these patients and remained unchanged or increased in each 14.3% (Figure 6).

## 4. Discussion

We saw a significant reduction of DME, independent of whether anti-VEGF or dexamethasone had been injected. The number of HF, as well as the number of HE, did not diminish clearly after either treatment. However, the percentage of eyes with a reduction in the number of foci at the end of the study period was as for the reduction of the CRT, slightly higher in the DEX group. There was also a trend towards a dynamic shift of the foci from the inner to the outer retinal layers. Fifteen percent of eyes showed an accumulation of larger diameter HF to visible HE over time, all treated with anti-VEGF agents, and the conversion of HF to HEs in these patients was independent of the change in macular fluid.

In the literature, various different hypotheses about the origin of HF have emerged that might in fact coexist. HF could be precursors of hard exudates, migrating RPE cells (in AMD), degenerated photoreceptor cells, or aggregations of activated immune cells, such as microglia. Bolz et al. first described such HF in 12 patients with DME using different OCT techniques [30]. These foci were interpreted as the morphologic sign of lipid extravasation obviously forming HEs, and they were not observable in classic examinations such as those using ophthalmoscopy or fundus photographs [30]. Retinal HEs are composed of lipid and proteinaceous material, such as fibrinogen and albumin that leak from the impaired BRB. They are deposited primarily in the OPL of the retina. Pemp et al. also demonstrated subretinal HF on OCT in patients with DME which may be associated with the future subfoveal deposition of HEs [23]. During the three-month treatment period, the HF in 24 eyes with DME, which were initially distributed through all retinal layers, shifted downwards and formed larger aggregates, although a rapid reduction in DME was seen in all patients [35]. When the aggregates reached a diameter of about 100 *μ*m in SD-OCT, HE appeared at corresponding locations of fundus photographs. A possible source of these deposits was thought to be microaneurysms, and the larger a vessel was in diameter, the more deposits could be observed at the vessel wall [23]. Similarly, in our study of a subgroup of patients, HF in outer retinal layers accumulated to visible HE during the follow-up period of one to two months after the treatment (Figure 6). During therapy, HE increased significantly in number and size in these patients, while DME resolved. Of note, the HF that accumulated were larger in size than the fine hyperreflective dots, which were still detectable before and after therapy (Figure 6). We think these larger HF correspond to microexudates similar to what has been postulated by Bolz et al. and Pemp et al. [23, 30], and that the small HF or hyperreflective dots could resemble cells of macrophage origin, that might aim to phagocytose the accumulated lipids and proteins. As such, their occurrence is independent of the state of fluid accumulation.

We did not find the large HF as precursors of exudates in a majority of our patients contrary to the study by Pemp et al. [23]. All patients in their study had severe DME, which did not respond to previous therapy with focal or grid laser therapy. Thus, a specific subgroup of recalcitrant DME patients was selected that differed from our study cohort in the matter that the exudative pathophysiology might have been predominant in their patients. Furthermore, in our study, we found HEs only in 54% of patients at baseline on SD-OCT images and CFPs. Similarly, Ota et al. also found HEs in 50% of their patients, but they used only CFPs for analysis [36]. Thus, HF do not form HE in all patients and therefore might represent a different entity than microexudates.

We found that the HF that evolved to HE are larger HF which are primarily found in the outer retina. As well as this, we detected HF scattered through all retina layers. Before therapy, this occurred even more frequently in the inner retinal layers in part in proximity to blood vessels (Figure 1). This is in line with some authors also suggesting that HF are a different entity than exudates, because the foci are distributed throughout all retinal layers, and thus, they are unrelated to microaneurysms as their source. In our study, ORL foci were especially found in foveal cystoid spaces (Figure 1), although HEs are rarely deposited in cystoid spaces. Moreover, the ELM, which corresponds to the adherent junction between Müller glial cells and photoreceptors, would restrict the migration of extravasated material and macromolecules into the outer retinal layers. Thus, HF might also represent a different entity such as inflammatory monocytic cells like activated microglia or macrophages. Microglia are the resident immune cells in the retina. Ramified (“resting”) microglia reside in the inner and outer plexiform layers where they continuously monitor their environment. When activated, they shift towards an amoeboid morphology [35], where they exhibit a larger cell body with shorter processes, they are therefore more likely to be detected as bright dots on SD-OCT. Both microglia and HF are also primarily found in the inner retina in DR and shift towards the outer retinal layers during DR progression, similar to what we found in our study (Figure 5). Case studies using histological techniques on retinal cross-sections revealed increasing numbers of moderately hypertrophic microglial cells in the plexiform layers of NPDR patients [37]. As DME arises, microglia have been observed in the outer retina and subretinal space [38]. In tissues from PDR patients, clusters of microglial cells could be found surrounding ischemic areas and new vessels together with a significant rise in their total number [38]. Furthermore, some authors insisted that HF are distinct in nature from lipoprotein exudates, because they detected HF in diabetic patients at the initial stages of DR and also in those without DR who had no detectable sign of a disrupted BRB on FA or without any sign of DME on OCT [33]. In addition, Lee et al. also observed a positive correlation between the level of soluble cluster of differentiation (CD) 14; a cytokine associated with the innate immune response expressed in microglia, monocytes, and macrophages; and the number of HF in the inner retina in DME patients [31].

Another fact that points towards monocytes is the fact that HE could regress after treatment, but very slowly, usually in a few months or at least many weeks after fluid reabsorption. This is different from the greater resolution of HF only one month after anti-inflammatory treatment compared to anti-VEGF therapy in our study. Therefore, we speculate that such a rapid response supports the fact that the fine HF that are distributed through all retinal layers in patients without visible HEs represent activated and swelled microglia as part of an inflammatory response.

As aforementioned, anti-VEGF agents, such as ranibizumab, have been successfully used to treat DME [39, 40]. Steroids are presumed to have more potent anti-inflammatory properties compared with anti-VEGF agents and have also been shown to effectively cause resolution of DME [14, 16]. Although complete resolution of DME may be achieved with both these treatment options, there may be several factors that predict the treatment response dependent on the agent used to treat the DME. In a study by Chatziralli, the reduction of the numbers of HF was not influenced by the choice of treatment option used to reduce the DME (ranibizumab or dexamethasone implant) [34]. In our study, there was a trend towards a greater reduction of HF in the DEX group, suggesting that inflammation and hyperpermeability with subsequent lipoprotein extravasation are potentially both implicated in the pathogenesis of HF and DME. Furthermore, VEGF has been demonstrated to induce microglial activation and anti-VEGF agents and, thus, also counteract this activation [41]. This might explain why there is not the obvious advantage of steroids over anti-VEGF in the reduction of the number of foci. In line with most studies that demonstrated a significant reduction of HF in DME and wet AMD patients after treatment [22, 34, 42–47], we did not find a significant reduction in the number of HF, along with a significant reduction in CRT. Most studies used a standardized three-time loading phase, and since postoperative observation in the present study took place two to four weeks after only one injection, no exact comparison is possible. It can be speculated that the reduction in HF numbers might have been greater if examination had been performed after three injections. Another explanation for the only slight decrease in the number of foci might be due to improve detection of hyperreflective foci/dots after resorption of edema. As the retinal architecture is restored, the foci are just easier to detect.

An important consideration is the fact that HF, among other OCT detectable morphological features, might serve as a better predictor of visual outcome in DME patients than the quantitative reduction of macular fluid as studies demonstrated only a moderate correlation of CRT with VA in DME patients [48, 49]. The integrity of the ellipsoid zone and the ELM has been shown to be the best predictor of visual outcome so far [20, 21]. We found HF as strong predictors of visual outcome in DME patients, demonstrated by a highly significant correlation of baseline HF (IRL and ORL foci) with baseline VA and final VA. This is in line with the literature on HF in DME patients [19, 22, 23]. Similar observations have also been reported in patients with age-related macular degeneration [44] and branch retinal vein occlusion [50]. Especially, HF in the outer layers correlated with poor visual function and with the disruption of the ELM and IS/OS line in patients with DME [51]. Hyperreflective foci, IS/OS, and ELM line disruption seems to represent outer blood-retinal barrier breakdown and photoreceptor dysfunction [51]. Fewer HF might represent better retinal tissue integrity, whereas the presence of many HF reflects tissue disintegration, a higher grade of inflammation, and more severe DME conditions. Since damaged ELM and/or IS/OS are not necessarily accompanied by macular edema in DME, this might be a good explanation for why visual acuity does not always improve after therapy in DME patients.

Further, we wanted to gain more insight into HF pathophysiology by performing OCTA in our patients. OCTA is a noninvasive imaging modality that enables a visualization of the retinal microvasculature. A comparative analysis of HF on SD-OCT and corresponding OCTA scans revealed that HF in the outer retinal layers were not attached to blood vessels in our patients. We identified them in spaces without any sign of vascularization, thus suggesting resident microglia as their origin, rather than exudation from leaking blood vessels. So far, only one other study evaluated HF on OCTA, but the authors looked only at HF in the INL and the Henle fiber layer (HFL), which is located at the border between ONL and OPL [52]. Murakami et al. found HF in the INL to be frequently attached to capillaries on OCTA images, which therefore might represent lipid-laden macrophages or precursors of HEs. In the HFL, HF were found to be in part enwrapped by a “reflectance decorrelation signal” [52]. They hypothesized that these HF contribute to photoreceptor damage and neuroglial dysfunction and might be of microglial/macrophage origin, because of the correlation between the “reflectance decorrelation signal” and visual impairment and disruption of the ellipsoid zone of the photoreceptors on OCT [52].

One important consideration is the image modality chosen to detect the HF, which has a potential effect on the detection rate. One study by Bolz et al. compared the detection rate of HF on three different OCT devices (two time-domain OCT devices, one SD-OCT) [30]. The study concluded that all three devices were able to detect the same HF in the same intraretinal locations; however, their features, density, and distribution throughout the retinal layers could be determined more precisely on SD-OCT, due to the higher resolution [30]. Furthermore, the eye-tracking system of the device offers the possibility of comparing the same retinal location in repeated examination. More recent OCT technologies include enhanced deep imaging OCT (EDI-OCT), which allows better visualization of the choroid and the swept-source (SS) OCT. This also allows a faster and more in detail visualization of the retinal and choroidal architecture and is less prone for artefacts due to media opacities or due to eye movement. A recent study found more HF on the Spectralis SD-OCT, the same device that we used for this study, compared to a SS-OCT device [53]. This was most likely due to the higher contrast delivered by the Spectralis SD-OCT [53]. Thus, currently, there is no obvious advantage of using the most recent SS-OCT technology when assessing OCT biomarkers like HF, apart from the shorter acquisition time and easier acquisition protocol. Most importantly, specialists should use the same device at every follow-up examination, when assessing HF or other biomarkers of treatment response.

The strengths of our study are its prospective design and the direct comparison of the two different treatment options for DME on the quality and quantity of HF. Limitations are the following: a relatively small sample size, short follow-up, and the fact that no histological samples were collected. Without histological correlation of the lesions on SD-OCT with immunohistochemistry markers for reactive RPE or microglial cells, we cannot definitively conclude on the origin of HF.

## 5. Conclusion

In conclusion, this is the first prospective study to comparatively study the quality and quantity of HF on OCT and OCTA in patients with DR, before and after treatment, with either primarily antiangiogenic or anti-inflammatory therapy. We assume HF to be related to microexudates in a subset of patients. These foci are larger in diameter on SD-OCT and found in outer retinal layers, whereas the fine hyperreflective dots, which are scattered through all retinal layers, correspond to monocytes such as microglia or macrophages.

We suggest that HF might predict treatment responses in patients with DME, as we could demonstrate a greater decrease in the number of HF in patients treated with dexamethasone implants compared to anti-VEGF agents. Further investigation is warranted to determine whether HF could serve as a biomarker to develop new endpoints for clinical imaging trials and more accurate disease monitoring in clinical practice. Further studies on HF should implement a longer follow-up period and automated, objective assessment of HF using specific software. This knowledge may contribute to the development of proper risk assessment, and therefore enable personalized decision making.

## Figures and Tables

**Table 1:** Baseline characteristics of 40 diabetic study patients.

No. of eyes (patients)	59 (51)
Male/female	39/12
Age, years	58,9 (±8)
Diabetic retinopathy stage	
Mild NPDR	7
Moderate NPDR	11
Severe NPDR	16
PDR	25
Intravitreal medication	
Bevacizumab	19
Ranibizumab	10
Aflibercept	11
Dexamethasone	19
DME type	
Focal	8
Diffuse:	51
Central retinal thickness, *μ*m	414.2 (±79.8)
No. hyperreflective foci	131.1 (±103)
No. hard exudates	13.9 (±18)
IS/OS disruption	
Yes	7
No	52

**Table 2:** Mean change in central retinal thickness and number of hyperreflective foci of 51 diabetic study patients at the baseline visit and during follow-up.

Group	Number of eyes (patients) visit 1	Number of eyes (patients) visit 2	Mean CRT + SD at baseline (*μ*m)	Mean CRT + SD at visit 1 (*μ*m) (^∗^*p* value)	Mean CRT + SD at visit 2 (*μ*m) (^∗^*p* value)	Mean number of foci + SD at baseline (*μ*m)	Mean number of foci + SD at visit 1 (*μ*m) (^∗^*p* value)	Mean number of foci + SD at visit 2 (*μ*m) (^∗^*p* value)
VEGF group	40 (35)	32 (27)	455.5 ± 139	402.5 ± 13 (*p* = 0.001)	380.8 ± 123 (*p* = 0.021)	130.6 ± 100	120.2±94 (*p* = 0.105)	111.1 ± 88 (*p* = 0.062)
DEX group		19 (16)	471.6 ± 112		381.9 ± 99 (*p* = 0.007)	123.4 ± 94		94.9 ± 89 (*p* = 0.020)

CRT: central retinal thickness; SD: standard deviation; visit 1: 15 days; visit 2: 30 days; ^∗^two-tailed *t*-test.

**Table 3:** Assessment percentage of eyes with a change in macular edema and change in the number of hyperreflective foci at the end of the treatment period.

Group	Macular edema				Number of foci	
	Dry	Decreased	Unchanged	Increased	Decreased	Increased
VEGF group	2/32 (6.25%)	22/32 (68.75%)	4/32 (12.5%)	4/32 (12.5%)	20/32 (62.5%)	12/32 (37.5%)
DEX group	8/19 (42%)	10/19 (53%)	1/19 (5%)	—	13/19 (68%)	6/19 (32%)

## Data Availability

All clinical data is available for the reader in the supplemental file.

## Supplementary Materials

Supplementary Materials Supplemental word file with an overview over the included study patients. Date of birth, gender, type of diabetic macular edema, disruption of inner segment/outer segment line, central retinal thickness, number and location of hyperreflective foci and hard exudates at baseline, and 15 and/or 30 days after therapy are shown.
