# Supplementary material for: Behavior of SD-OCT Detectable Hyperreflective Foci in Diabetic Macular Edema Patients after Therapy with Anti-VEGF Agents and Dexamethasone Implants
Source: J Diabetes Res. 2021 Apr 13;2021:8820216. doi: 10.1155/2021/8820216 (PMC8060103; doi:10.1155/2021/8820216)
Supplement: Supplementary Materials — Supplemental word file with an overview over the included study patients. Date of birth, gender, type of diabetic macular edema, disruption of inner segment/outer segment line, central retinal thickness, number and location of hyperreflective foci and hard exudates at baseline, and 15 and/or 30 days after therapy are shown. [file 8820216.f1.docx]

**Supplemental file to: Behavior of SD-OCT detectable hyperreflective foci in diabetic macular edema patients after therapy with anti-VEGF agents and dexamethasone implants**

**Anne Rübsam^1,2^, Laura Wernecke^1^, Dominika Pohlmann^1,2^, Bert Müller^1^, Oliver Zeitz^1,2^, Antonia M. Joussen^1,2^**

^1^ Department of Ophthalmology, Charité University Medicine Berlin, corporate member of Freie Universität Berlin, Humboldt-Universität zu Berlin, and Berlin Institute of Health

^2^ Berlin Institute of Health (BIH), Berlin, Germany

e-mail: anne.ruebsam@charite.de

| **patient** | **eye** | **Medikament** | **DR stage** | **IS/OS desintegration pre/after IVI** | **HF baseline IVI total** | **HF inner layer baseline IVI** | **HF outer layer baseline IVI** | **DME type** | **HF post IVI total** | **HF inner layer post IVI** | **HF outer layer post IVI** | **CRT pre IVI** | **CRT post IVI** | **edema resolution** | **HE pre/post IVI** |
| --- | --- | --- | --- | --- | --- | --- | --- | --- | --- | --- | --- | --- | --- | --- | --- |
| MJ *2.05.61  30 days | OD | Avastin | PDR | No/no | 109 | 66 | 43 | diffuse | 27  63 | 8  42 | 13  21 | (464) | (259)260 | Reduced  reduced | 0/0  0 |
| AR *30.05.64  30 days: | OD | Avastin | PDR | No/no | 63 | 42 | 21 | diffuse | 69  42 | 37  36 | 32  6 | (260) | (253)  278 | Reduced  reduced | 0/0  0 |
| CK *08.03.70  30 days: | OD | Avastin | PDR | No/no | 42 | 36 | 6 | diffuse | 38  41 | 32  35 | 7  6 | (278) | (253)  257 | Dry  dry | 0/0  0 |
| SJ *25.10.68  30 days: | Od | Avastin | PDR | No/no | 41 | 35 | 6 | diffuse | 40  51 | 25  34 | 15  17 | (257) | (249)  297 | Dry  increased | 0/0  0 |
| BW *5.10.1953  30 days: | OD | Eylea | PDR | Yes/yes | 125 | 23 | 101 | diffuse | 178  104 | 31  32 | 147  72 | (682) | (429)  388 | Reduced  reduced | 8/11  9 |
| KMW *4.03.55 | OD | Eylea | PDR | No/no | 317 | 175 | 142 | diffuse | 274 | 99 | 175 | (551) | (494) | reduced | 17/13 |
| SW *30.12.50  30 days: | OD | Eylea | PDR |  | 302 | 71 | 231 | diffuse | 194  304 | 50  68 | 189  236 | (571) | (552)  419 | Reduced  reduced | 5/13  25 |
| DR *29.04.80 | OD | Ozurdex | PDR | No/no | 308 | 155 | 153 | diffuse | 201 | 143 | 228 | (706) | (676) | reduced | 15/15 |
| MK *09.06.76  30 days: | OD | Eylea | PDR | No/no | 323 | 117 | 206 | diffuse | 361  361 | 107  107 | 254  254 | (743) | (573)  573 | Reduced  reduced | 2/1  3 |
| CR  *1.01.1964  30 days: | OD | Eylea | PDR | No/no | 344 | 98 | 246 | diffuse | 324  302 | 77  71 | 247  231 | (574) | (569)  571 | Reduced  unchanged | 9/3  5 |
| OK *4.12.1966  30 days: | OS | Eylea | PDR | No/no | 125 | 48 | 77 | diffuse | 104  104 | 32  32 | 72  72 | (560) | (388)  388 | Reduced  reduced | 7/8  8 |
| UW *30.03.1965  30 days: | OS | Eylea | PDr | No/no | 104 | 32 | 72 | diffuse | 106  106 | 37  37 | 69  59 | (388) | (382)  382 | Reduced  reduced | 8/9  9 |
| KU *14.01.1978  30 days: | OS | Eylea | PDR | No/no | 119 | 45 | 74 | diffuse | 99  91 | 12  12 | 87  79 | (382) | (374)  313 | Reduced  reduced | 18/14  2 |
| PW  *23.12.1943  30 days: | OS | Lucentis | moderate NPDR | No/no | 85 | 10 | 75 | focal | 105  105 | 16  16 | 89  89 | (336) | (279)  279 | Dry  dry | 0/3  3 |
| AM *13.03.1959  30 dys: | OS | Lucentis | moderate NPDR | No/no | 131 | 55 | 76 | focal | 119  119 | 44  44 | 75  75 | (326) | (317)  317 | Reduced  reduced | 0/0  0 |
| DK *1.01.1970 | OD | Lucentis | severe NPDR | No/no | 128 | 20 | 108 | diffuse | 141 | 26 | 114 | (481) | (439) | reduced | 14/6 |
| YK *25.09.1961 | Od | Lucentis | severe NPDR | No/no | 259 | 70 | 189 | diffuse | 249 | 59 | 190 | (473) | (444) | reduced | 32/40 |
| MWK *13.11.1969 | OD | Lucentis | severe NPDR | No/no | 330 | 132 | 198 | diffuse | 339 | 52 | 287 | (551) | (542) | reduced | 0/5 |
| SR  *25.03.1963 | OD | Lucentis | severe NPDR | Yes/yes | 17 | 10 | 8 | diffuse | 2 | 2 | 0 | (624) | (387) | reduced | 0/0 |
| WA *12.11.1970 | OD | Avastin | moderate NPDR | No/no | 175 | 68 | 108 | diffuse | 135 | 45 | 90 | (291) | (387) | unchanged | 88/70 |
| BG  *01.06.1965 | OD | Ozurdex | moderate NPDR | No/no | 90 | 31 | 60 | diffuse | 106 | 30 | 75 | (564) | (452) | reduced | 3/3 |
| BG | OS | Ozurdex | moderate NPDR | No/no | 128 | 40 | 88 | diffuse | 204 | 57 | 150 | (639) | (505) | reduced | 3/2 |
| YW *18.03.1959  30 days | Od | Avastin | moderate NPDR | No/no | 58 | 16 | 41 | diffuse | 77  86 | 21  16 | 54  70 | (595) | (230)  338 | Reduced  reduced | 0/0  0 |
| AM *23.11.1965  30 days: | OD | Avastin | moderate NPDR | No/no | 86 | 16 | 70 | diffuse | 79  57 | 7  11 | 72  46 | (338) | (329)  347 | Reduced  increased | 0/0  0 |
| NS *30.10.1950  30 days: | OD | Avastin | moderate NPDR | No/no | 57 | 11 | 46 | diffuse | 82  59 | 15  8 | 67  51 | (347) | (263)  291 | Reduced  reduced | 0/0  0 |
| NS *30.10.1950  30 days: | OS | Avastin | moderate NPDR | No/no | 82 | 20 | 62 | diffuse | 63  100 | 19  23 | 44  77 | (381) | (331)  348 | Unchanged  reduced | 6/3  0 |
| GK *22.08.1959  30 days: | OS | Avastin | Moderate NPDR | No/no | 100 | 23 | 77 | diffuse | 80  91 | 11  12 | 69  79 | (348) | (344)  313 | Unchanged  reduced | 0/1  2 |
| WFP *27.05.1951  30 days: | OD | Avastin | mild NPDR | No/no | 87 | 41 | 46 | focal | 89  89 | 39  39 | 50  50 | (416) | (384)  384 | Reduced  reduced | 18/15  15 |
| WFP  30 days: | OS | Avastin | mild NPDR | No/no | 202 | 57 | 146 | diffuse | 200  200 | 49  49 | 163  163 | (326) | (312)  312 | Unchanged  unchanged | 62/69  69 |
| RM *22.09.1965 | OD | Ozurdex | PDR | No/no | 132 | 63 | 66 | diffuse | 75 | 18 | 59 | (525) | (301) | dry | 0/2 |
| AK *16.09.1966 | OD | Ozurdex | PDR | No/no | 99 | 24 | 75 | diffuse | 60 | 8 | 52 | (464) | (343) | reduced | 1/0 |
| YW *1.12.2959 | OD | Ozurdex | PDR | No/no | 47 | 23 | 24 | diffuse | 18 | 3 | 6 | (367) | (347) | reduced | 0/0 |
| AN  *30.11.1963  30 days: | OD | Lucentis | mild NPDR | No/no | 90 | 29 | 61 | focal | 68  68 | 14  14 | 63  63 | (249) | (247)  247 | Reduced  reduced | 24/22  22 |
| FH  *28.12.41  30 days: | OS | Lucentis | PDR | No/no | 20 | 9 | 11 | diffuse | 10  10 | 2  2 | 8  8 | (432) | (400)  400 | Reduced  reduced | 0/0  0 |
| UM  *29.04.1965  30 days: | OS | Avastin | severe NPDR | No/no | 79 | 26 | 53 | diffuse | 93  93 | 40  40 | 53  53 | (373) | (389  389) | Increased  increased | 3/11  11 |
| UM  30 days: | OD | Avastin | severe NPDR | Yes/yes | 141 | 24 | 139 | diffuse | 134  134 | 37  37 | 97  97 | (325) | (341)  341 | Reduced  reduced | 33/29  29 |
| MT *15.04.1967  30 days: | OD | Avastin | PDR | No/no | 79 | 26 | 53 | diffuse | 69  69 | 25  25 | 44  44 | (468) | (465)  465 | Unchanged  unchanged | 0/0  0 |
| NBD  *15.11.61  30 days: | OD | Avastin | PDR | No/no | 69 | 25 | 44 | diffuse | 80  80 | 26  26 | 54  54 | (456) | (413)  413 | Reduced  reduced | 0/0  0 |
| CK *30.06.1978  30 days: | OD | Eylea | mild NPDR | No/no | 23 | 8 | 15 | diffuse | 27  27 | 10  10 | 17  17 | (413) | (399)  399 | Reduced  reduced | 0/0  0 |
| SC  *16.05.1963  30 days: | OD | Eylea | mild NPDR | No/no | 17 | 9 | 8 | diffuse | 14  14 | 2  2 | 12  12 | (395) | (406)  406 | Increased  increased | 0/0  0 |
| SC  30 days: | OS | Eylea | moderate NPDR | No/no | 120 | 16 | 104 | diffuse | 106  106 | 17  17 | 89  89 | (407) | (376)  376 | Reduced  reduced | 22/9  9 |
| SM  *30.08.54 | OD | Lucentis | severe NPDR | No/no | 19 | 16 | 3 | focal | 12  12 | 11  11 | 1  1 | (389) | (360)  360 | Reduced  reduced | 0/0  0 |
| SM  30 days: | OS | Lucentis | severe NPDR | No/no | 107 | 27 | 80 | diffuse | 89  65 | 14  9 | 75  54 | (682) | (469)  346 | Reduced  reduced | 2/1  2 |
| HH  *1.12.1965  30 days: | OD | Avastin | mild NPDR | No/no | 148 | 57 | 91 | diffuse | 124  124 | 38  38 | 86  86 | (452) | (483)  483 | Increased  increased | 31/14  14 |
| ÜB  *01.01.60  30 days: | OS | Avastin | PDR | Yes/yes | 373 | 124 | 249 | diffuse | 284  284 | 87  87 | 197  197 | (768) | (896)  896 | Increased  increased | 22/17  17 |
|  |  |  |  |  |  |  |  |  |  |  |  |  |  |  |  |
